# Reduction of Advanced-Glycation End Products Levels and Inhibition of RAGE Signaling Decreases Rat Vascular Calcification Induced by Diabetes

**DOI:** 10.1371/journal.pone.0085922

**Published:** 2014-01-21

**Authors:** Mathieu R. Brodeur, Céline Bouvet, Sonia Bouchard, Simon Moreau, Jeanne Leblond, Denis deBlois, Pierre Moreau

**Affiliations:** Faculty of Pharmacy, Université de Montréal, Montréal, Québec, Canada; University of Pittsburgh, United States of America

## Abstract

Advanced-glycation end products (AGEs) were recently implicated in vascular calcification, through a process mediated by RAGE (receptor for AGEs). Although a correlation between AGEs levels and vascular calcification was established, there is no evidence that reducing *in vivo* AGEs deposition or inhibiting AGEs-RAGE signaling pathways can decrease medial calcification. We evaluated the impact of inhibiting AGEs formation by pyridoxamine or elimination of AGEs by alagebrium on diabetic medial calcification. We also evaluated if the inhibition of AGEs-RAGE signaling pathways can prevent calcification. Rats were fed a high fat diet during 2 months before receiving a low dose of streptozotocin. Then, calcification was induced with warfarin. Pyridoxamine was administered at the beginning of warfarin treatment while alagebrium was administered 3 weeks after the beginning of warfarin treatment. Results demonstrate that AGEs inhibitors prevent the time-dependent accumulation of AGEs in femoral arteries of diabetic rats. This effect was accompanied by a reduced diabetes-accelerated calcification. *Ex vivo* experiments showed that N-methylpyridinium, an agonist of RAGE, induced calcification of diabetic femoral arteries, a process inhibited by antioxidants and different inhibitors of signaling pathways associated to RAGE activation. The physiological importance of oxidative stress was demonstrated by the reduction of femoral artery calcification in diabetic rats treated with apocynin, an inhibitor of reactive oxygen species production. We demonstrated that AGE inhibitors prevent or limit medial calcification. We also showed that diabetes-accelerated calcification is prevented by antioxidants. Thus, inhibiting the association of AGE-RAGE or the downstream signaling reduced medial calcification in diabetes.

## Introduction

Vascular aging is related to a progressive stiffening of large arteries [1] and an acceleration of vascular stiffness has been demonstrated in type 2 diabetic patients [2]. This stiffening of vessel walls is caused by a pathological remodeling process that includes elastin fragmentation, collagen deposition, matrix cross-linking and elastocalcinosis [3]. Elastocalcinosis is different from the calcification associated with atherosclerosis. It localizes on elastin lamellae and does not involve lipids or inflammatory cell infiltration. Numerous studies demonstrated that medial calcification is accelerated by diabetes [4]–[6] and, in diabetic patients, elastocalcinosis has also been associated with peripheral artery disease [7]. Moreover, it was shown that, in patients suffering from diabetes, medial calcification of peripheral arteries is a strong independent predictor of cardiovascular mortality and morbidity [8]. It is now well established that vessel wall mineralization is highly cell-regulated. Indeed, an *in vivo* study demonstrated that vascular smooth muscle cells (VSMC) undergo phenotypic transdifferentiation into osteogenic-like cells able to mineralize [9]. In accordance, VSMC from calcified distal peripheral arteries of diabetic patients have an osteocytic/chondrocytic phenotype [10]. Despite this strong association between elastocalcinosis and diabetes, the mechanism leading to medial calcium accumulation in diabetes remains unknown.

Advanced-glycation end products (AGEs) result from a reaction between a lysine or a hydroxylysine of a protein and a sugar, producing the Maillard reaction, rearranged in a more stable Amadori product [11]. Thus, hyperglycemia in diabetes enhances the formation of AGEs and leads to fluorescent or non-fluorescent products, sometimes creating cross-links [12]. Because AGEs cross-links disappear only with protein turnover, an accumulation of AGEs is particularly observed in proteins with a long biological half-life such as collagen or elastin [13], [14]. Some studies have established a correlation between AGEs levels and vascular calcification [15], [16]. Of particular interest, Bruël *et al*. demonstrated that AGEs accumulation in the vascular wall was correlated with age-related aortic stiffness in rats [13]. Furthermore, elastin stiffness induced by AGEs cross-links appeared in parallel with an increase of calcium ion uptake by elastin [17]. Several treatments targeting AGEs have also demonstrated improvement of arterial stiffening. For example, inhibitors of AGEs formation, such as aminoguanidine, prevent age-related arterial stiffening [18] and alagebrium, a breaker of AGEs, improves arterial compliance in aged patients with vascular stiffness [19]. Thus, these results suggest that AGEs could be implicated in vascular calcification and thus, drugs modulating AGEs levels could reduce arterial stiffness by reducing calcification.

Recently, several studies using different models of VSMC demonstrated the implication of AGEs in calcification. Indeed, it was shown that exposure of VSMC to AGEs induced cell calcification. This effect required the interaction of AGEs with the receptor for advanced glycation end-products (RAGE) since addition of a neutralizing antibody targeting this receptor inhibited the calcification [20], [21]. Similarly, it was found that diabetic serum induced a RAGE-dependent calcification of VSMC [21]. It was also demonstrated that RAGE activation caused the osteogenic differentiation of VSMC by the activation of the ERK1/2 pathways [22]. However, the implication of ERK1/2 is contradicted by the Tanaka group who demonstrated that p38 is the mitogen-activated protein (MAP) kinase responsible for the calcification induced by RAGE [21]. Moreover, *in vitro* evidence suggests that induction of NADPH oxidase activity by AGEs is implicated in the RAGE-dependent calcification [23], [24]. Despite all this *in vitro* information, there is, to our knowledge, no study confirming that AGEs formed under diabetic conditions induce *in vivo* medial calcification.

The present study was designed to determine if AGEs are essential for the development of arterial calcification in diabetes and if RAGE signaling plays an important role in that pathological process. In order to study the mechanisms involved in the acceleration of medial calcification by diabetes, we used a rat experimental model developed in our laboratory [25]. In our model, diabetes is induced by a high fat diet and a low dose of streptozotocin, while medial calcification is triggered by warfarin/vitamin K treatment. We found that preventing the accumulation of AGEs or reducing AGEs levels with pyridoxamine or alagebrium reduced diabetes-accelerated medial calcification. Our results also suggest that inhibition of RAGE signaling reduced the calcification induced by AGEs.

## Materials and Methods

### *In vivo* experiments

#### Ethics statement

All animal experiments were approved by the Animal Care and Use Committee of Université de Montréal with the guide for the care and use of laboratory animals published by the US National Institutes of Health. All surgery was performed under sodium pentobarbital anesthesia, and all efforts were made to minimize suffering.

#### Treatments

Male Wistar rats (initial weight of 50–75 g) were obtained from Charles River Breeding Laboratories (St-Constant, Qc, Canada). They were fed a high fat diet containing 45 kcal % fat, 35 kcal % carbohydrates and 20 kcal % protein (Research diets, D12451, New Brunswick, NJ, USA) *ad libitum* during 8 weeks, followed by a single dose of streptozotocin (STZ, 30 mg/kg intra-peritoneally). Four weeks after the injection of STZ, rats received warfarin (20 mg. kg^−1^. day^−1^ in drinking water) and vitamin K (phylloquinone, 15 mg. kg^−1^. day^−1^ sub-cutaneous injection, Spectrum chemical, New Brunswick, NJ, USA) during 3 (D_3_), 5 (D_5_) and 7 (D_7_) weeks.

**Figure 1:**
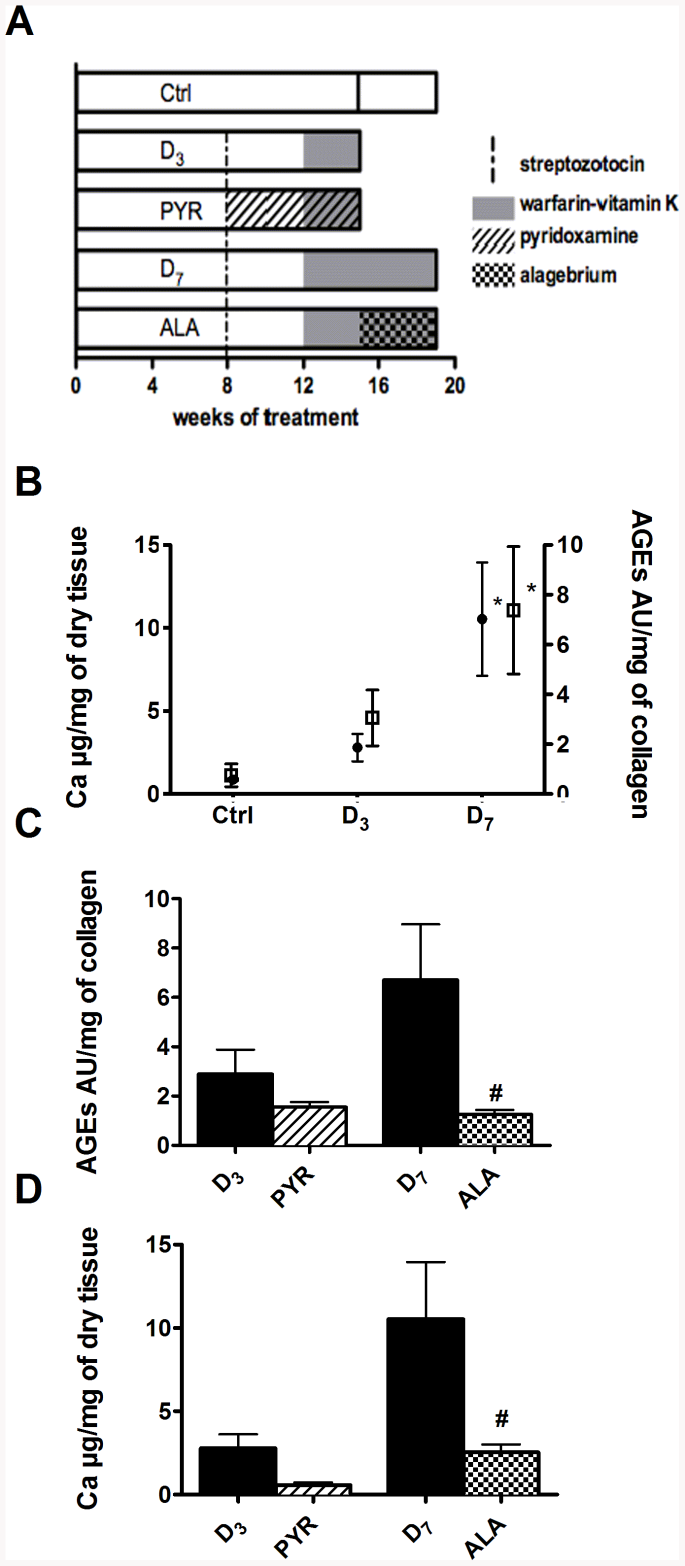
Implication of AGEs in diabetes-accelerated femoral calcification in vivo. (A) Treatment scheme. Open bars represent a high fat diet, diagonals represent the pyridoxamine treatment while white and black squares are for the alagebrium treatment. The gray bars show the warfarin/vitamin K treatment and the number exponent on D indicates the duration in week of WVK treatment. The vertical dashed lines represent the injection of streptozotocin. (B) Quantification of fluorescent advanced glycation end-products (open squares) and calcium (closed circles) accumulated in femoral vascular wall. (* p<0.05 vs Ctrl). Effect of pyridoxamine (PYR) and alagebrium (ALA) on (C) AGEs and (D) calcium accumulation in femoral arteries. (# p<0.05 vs D7). For convenience and because no hemodynamic differences were observed, the two control groups were pooled for the experiments presented in panel B.

To determine the implication of AGEs in initiating elastocalcinosis, a subgroup of D_3_ rats received pyridoxamine (200 mg. kg^−1^. day^−1^) in powdered chow starting the same day as the STZ injection (thus during 7 weeks, including the 3 weeks of warfarin vitamin K (WVK) treatment) to prevent AGEs formation (group labeled PYR). To study the role of AGEs later in the calcification process, alagebrium (10 mg. kg^−1^. day^−1^, Synvista, Montvale, NJ, USA) was introduced in the food 7 weeks after the STZ injection (after 3 weeks of WVK treatment) and rats studied 4 weeks later (group labeled ALA). Dosages were adjusted every second day according to food intake. Controls consisted of age-matched untreated rats (Ctrl_3_ or Ctrl_7_) (**Figure 1A**). Rats also received three different antioxidants, in powdered chow starting the same day as the WVK treatment. Thus, rats were treated during 3 or 5 weeks with alpha-lipoïc acid (100 mg. kg^−1^. day^−1^, LIP group), 4-hydroxy-tempol (20 mg. kg^−1^. day^−1^, TEM group) or apocynin (2,5 mg. kg^−1^. day^−1^, APO group).

#### Hemodynamic and metabolic parameters

Animals were anesthetized with pentobarbital (65 mg/kg, Ceva Santé Animale, Libourne, France) and short catheters (polyethylene-50, approx. 10 cm, Folioplast SA, Sarcelles, France) were inserted into the distal abdominal aorta through the left femoral artery and into the aortic arch through the left carotid. Catheters were connected to a pressure transducer to allow the measurement of systolic and diastolic blood pressures at each location as previously described [26]. Glycemia was quantified on an arterial blood sample with a glucometer (AccuSoft Advantage, Roche, Laval, Qc, Canada). The aorta and femoral artery were harvested, after sacrifice (exsanguinations under pentobarbital anesthesia). Portions were frozen at −80°C for calcium levels, AGEs quantification and superoxide anion evaluation.

#### Advanced glycation end-products measurement

Glycation of collagen was measured as previously described [27]. This method uses the fluorescent properties of some AGEs to evaluate their formation in the vascular wall. Collagen contains a high concentration of amino acid hydroxyproline, up to 14% of the dry weight of collagen, and this amino acid is measured to estimate the collagen content. After their pulverization at −80°C, vascular tissues were suspended in PBS and rinsed in distilled water (centrifugation at 10 000 rpm during 15 minutes). The extraction of lipids was performed with chloroform-methanol (2∶1) overnight at room temperature. Then, the samples were rinsed successively in methanol, distilled water and HEPES buffer 0.02 M. The pellets were resuspended in 0.5 mL of HEPES buffer. Blanks were prepared with known amounts of collagen. One hundred units of bacterial type VII collagenase were added to each sample and the digestion was allowed to proceed during 24 hours at 37°C with constant agitation. The supernatants, obtained after centrifugation at 3 000 rpm for 3 minutes, were used for determination of hydroxyproline content and fluorescence of AGEs. In order to measure hydroxyproline, the supernatant was hydrolyzed by 2N sodium hydroxide and by autoclaving. Chloramine-T reagent was added for 25 minutes at room temperature for oxidation. Finally, Ehrlich's reagent was added and the chromophore was developed by incubation at 65°C during 20 min. Absorbance was read at 550 nm. This technique allowed the measurement of the hydroxyproline content of the samples (standards of hydroxyproline) and their collagen content. Fluorescence of the supernatants for AGEs was measured by excitation at 370 nm and emission at 440 nm. Results of glycation were expressed as arbitrary units per mg of collagen. As noted by Monnier *et al*. [27] fluorescence at 440 nm was associated with molecules derived from collagen, but could also derive from other sources. However, no more specific assays are presently available to evaluate the source of fluorescence.

#### Detection of superoxide anion

Superoxide anion was measured using the lucigenin enhanced chemiluminescence method. Krebs-HEPES buffer (saturated with 95% O_2_ and 5% CO_2_ at room temperature during 30 minutes) was incubated in 96-well plate with lucigenin 5 µmol/L during 20 minutes in the dark. This concentration of lucigenin has been shown to accurately reflect the levels of ambient O2•- and is not subject to the redox cycling and artificial production of superoxide observed with higher concentrations of the agent [28] [29]. Background counts were registered during 5 minutes by a Microbeta Trilux. Meanwhile, 5 mm aortic rings were incubated in Krebs-HEPES buffer during 10 minutes in the dark at 37°C. Aortic rings were placed in wells. Then, chemiluminescence was read every minute during 10 minutes. Finally, aortic rings were dried. Lucigenin counts (mean of counts minus mean of background counts) were expressed as cpm/mg of dry tissue. The chemical specificity of this light-yielding reaction for superoxide anion has been reported previously [30].

### *Ex vivo* experiments

#### N-methylpyridinium

N-methylpyridinium (N-m) synthesis was realized according to the protocol of Zill *et al*. [31]. The product was analyzed by ^1^H NMR (DMSO-d_6_) δ (ppm) 4.35 (3H, s, CH3), 8.13 (2H, t, J = 7.0 Hz, CH_meta_), 8.58 (1H, t, J = 8.1 Hz, CH_para_), 8.99 (2H, d, J = 5.7 Hz, CH_ortho_) and ^13^C (DMSO-d_6_) δ (ppm) 49.25 (CH_3_), 128.99 (C_meta_), 146.37 (C_ortho_), 146.92 (C_para_). NMR spectra were recorded on a Bruker Avance 300 MHz (QNP probe).

#### Arterial culture

Male Wistar rats (initial weight 125–150 g) obtained from Charles River Breeding Laboratories (St-Constant, Qc, Canada) received STZ (60 mg/kg, i.-p. injection) to destroy pancreatic cells. Once β-cells destruction confirmed (measurement of glycemia two days after the injection of STZ), an insulin implant was inserted under the skin to maintain glycemia around 15–20 mmol/L. This controlled hyperglycemia allows the *in vivo* formation of AGEs, while improving the survival of the rats. Glycemia and body weight were monitored twice a week. Eight weeks after the injection of STZ, rats were sacrificed by injection of pentobarbital as previously described. The femoral arteries were harvested and placed in culture.

#### Calcification

Femoral arteries of untreated and diabetic rats were placed into DMEM referred as the control condition (Ctrl). To induce calcification in a subset of these arteries, 10 µmol/L of warfarin was added to the medium and the concentration of PO_4_^3−^ was increased to 3.8 mmol/L two days after the addition of warfarin. Arterial culture in these calcification medium (CM) conditions was maintained during 4 days. To determine the implication of RAGE in elastocalcinosis, some femoral arteries were incubated with N-methylpyridinium (18 µg/mL). Three different anti-oxidants were used: 4-hydroxy-tempol, alpha-lipoïc acid and apocynin.

#### Calcium content

To measure calcium content, sections of arteries were dried at 55°C and calcium was extracted with 10% formic acid (30 µL/mg of dry tissue) overnight at 4°C. The colorimetric quantification was achieved through a reaction with o-cresolphtalein (Teco Diagnostics, Anaheim, CA, USA).

### *In vitro* experiments

#### Primary rat vascular smooth muscle cell culture

Aortic smooth muscle cells were isolated from aortas of untreated Wistar rats. After removing the endothelial cells and fibroblasts, the aorta was placed in DMEM containing 2 mg/mL collagenase type I (Sigma, St. Louis, MO, USA) and 0.1 mg/mL elastase type I (Sigma) and incubated for 30 minutes at 37°C. The adventitia was cleanly stripped off and the remaining medial tissue was placed in DMEM containing 6 mg/mL collagenase and 0.1 mg/mL elastase type I for 2 hours at 37°C with gentle agitation in order to obtain single cells and small cell clumps. The suspension of cells was centrifuged at 150×g for 10 minutes and resuspended in DMEM supplemented with 10% fetal bovine serum (FBS), 100 units/mL penicillin and 100 µg/mL streptomycin. Cells were cultured in 5% CO_2_ at 37°C and were harvested once a week with trypsin-EDTA. The cultured cells were morphologically elongated and stained positively with anti-alpha-smooth muscle actin monoclonal antibody. Cultures used in the present study were from the 4^th^ to 9^th^ passages.

#### Immunoblotting P-ERK and ERK

To verify that N-methylpyridinium was a ligand of RAGE, VSMCs were seeded in 12-well plates at 80% of confluence. Cells were stopped during 1 hour with 50% DMEM, 50% F12, 0,1% BSA Frachen V, 5 µg/mL transferring, 15 mmol/L HEPES 7.4, before stimulation with N-methylpyridinium in DMEM during 2 to 30 minutes. The experiment was performed again with a neutralizing anti-RAGE antibody or the corresponding non-specific IgG (R&D Systems, Minneapolis, MN, USA). P-ERK and ERK expressions (signaling pathway of RAGE) were evaluated by immunoblotting.

Total proteins were extracted in cold lysis buffer (150 mmol/L NaCl, 50 mmol/L Tris at pH 7.4, 1 mmol/L EDTA, 1 mmol/L EGTA, 0.5% Igepal, 1 mmol/L sodium ortho-vanadate, 0.2 mmol/L PMSF, 2 µg/mL aprotinin, 1 µg/mL pepstatin and 1 µg/mL leupeptin). Equal amounts of proteins (30 µg) were resolved by electrophoresis on 10% SDS-PAGE and transferred to nitrocellulose membranes. Nitrocellulose membranes were incubated overnight at 4°C with anti-phospho-ERK and anti-ERK antibodies (Cell signaling, Boston, MA, USA) followed by enhanced chemiluminescence detection using Typhoon scanner 9410.

### Drugs and statistical analysis

All drugs were purchased from Sigma Chemical Co. (Oakville, On, Canada) unless otherwise specified. Values are expressed as mean ± SEM. An analysis of variance followed by Bonferonni's correction was used to compare the groups (P had to be smaller than 0.05/number of comparisons to reach statistical significance).

## Results

**Figure 2:**
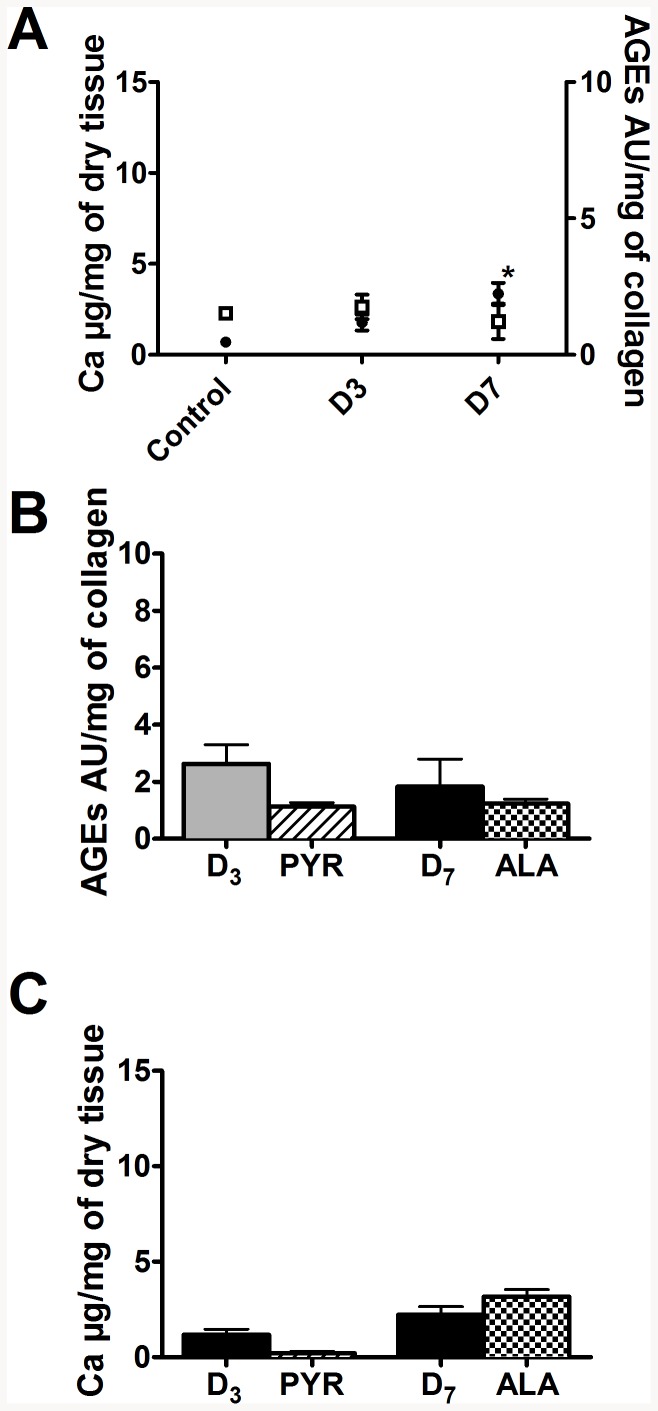
Implication of AGEs in diabetes-accelerated aortic calcification in vivo. (A) Quantification of fluorescent advanced glycation end-products (open squares) and calcium (closed circles) accumulated in the aortic wall. (* p<0.05 vs Ctrl). Effect of pyridoxamine (PYR) and alagebrium (ALA) on (B) AGEs and (C) calcium accumulation in aortae. For convenience and because no hemodynamic differences were observed, the two control groups were pooled for the experiments presented in panel A.

Initially, we wanted to evaluate if direct inhibition of AGEs production or elimination of accumulated AGEs could reduce medial calcification *in vivo*. To this end, we used the animal model of diabetes-accelerated medial calcification developed in our laboratory [25]. In this model, three weeks of WVK treatment induces calcification only in the presence of diabetes, indicating that some metabolic disturbances caused by diabetes accelerate calcification. All groups of rats receiving a high fat diet and a low dose of streptozotocin demonstrated a significant hyperglycemia, confirming the induction of diabetes (**Table 1**). The femoral calcification progressively increased with the duration of warfarin-vitamin K (WVK) treatment and diabetes, with significantly increased levels in the D_7_ group (**Figure 1B**). Furthermore, we observed a progressive accumulation of fluorescent AGEs in the femoral vascular wall (**Figure 1B**). Meanwhile, aortic calcium content increased slightly with the duration of WVK treatment and diabetes, reaching statistical significance in the D_7_ group (**Figure 2A**). Levels of calcification were 4.7 times higher in femoral arteries than in the aorta and AGEs accumulation was 3.7 times higher in femoral arteries than in the aorta where the levels were not significantly increased (**Figure 2A**). No significant changes of hemodynamic parameters were observed with the development of vascular calcification in the diabetic rats (**Table 1**).

**Table 1:** Anti-AGEs treatments: metabolic and hemodynamic parameters.

	GROUP					
Parameters	Ctr_3_	D_3_	PYR	Ctrl_7_	D_7_	ALA
**n = **	10	8	8	9	9	7
**Body weight (g)**	448±3	492±28	527±20	512±11	550±25	627±36
**Glycemia (mmol/L)**	4.2±0.4	14.2±1.9*	18.6±2.3	4.8±0.3	13.3±1.9*	12.2±1.9
**MBP_C_ (mmHg)**	112.4±6.5	125.0±5.6	147.2±4.8^a^	132.7±4.4	129.2±3.8	132.7±6.1
**PWV (m/s)**	6.5±0.4	5.7±0.3	6.6±0.2	6.2±0.3	6.4±0.1	6.2±0.2

MBP_C_: carotid mean blood pressure; PWV: pulse wave velocity; See methods for group description. *: P<0.05 vs respective control (Ctrl); ^a^: P<0.05 vs respective D group. ANOVA followed by Bonferroni's correction for multiple comparisons.

Treatments with modifiers of AGEs accumulation had a significant impact on the vascular wall. Pyridoxamine (PYR) prevented AGE accumulation on collagen (55% of reduction in the aorta and 45% in femoral arteries compared to D_3_) and calcium accumulation (around 80% of reduction) in femoral arteries (**Figures 1C and 1D**) and the aorta (**Figures 2B and 2C**). However, the reductions were not statistically significant, since the alterations themselves were modest at this time. Surprisingly, pyridoxamine was associated with a significant elevation of mean blood pressures (**Table 1**). Alagebrium (ALA) significantly reduced the fluorescence of AGEs and calcium accumulation in femoral arteries (**Figures 1C and 1D**). In contrast, it induced no significant change of calcium and AGEs contents in the aorta (**Figures 2B and 2C**).

**Figure 3:**
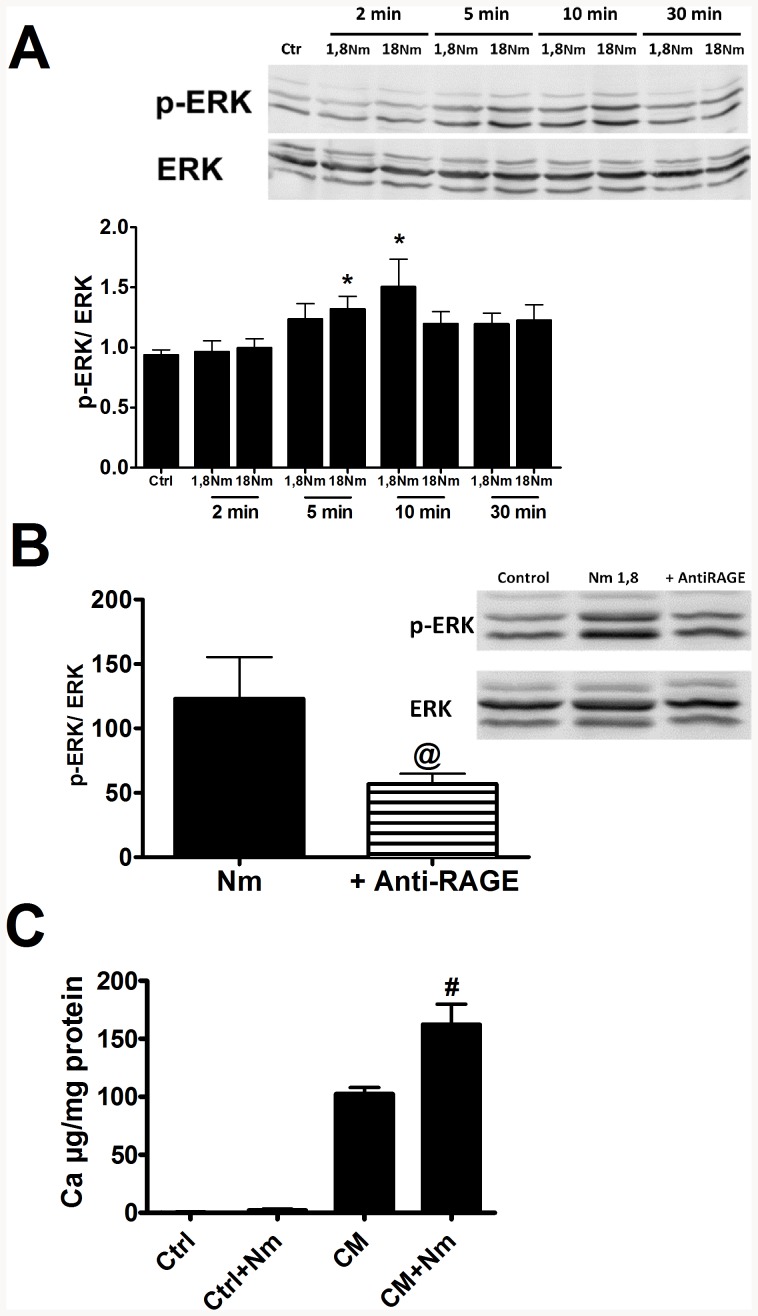
Impact of N-methylpyridinium on VSMCs. (A) P-ERK/ERK expression ratio after incubation with N-methylpyridinium at two concentrations (1.8 and 18 µg/mL) during 2 to 30 minutes (* p<0.05 vs Ctrl). (B) P-ERK/ERK expression ratio after 10-minute incubation with N-methylpyridinium (1,8 µg/mL) with or without anti-RAGE antibody (@ p<0.05 vs N-m 1,8, unpaired t test). (**C**) Enhancement of calcification by N-methylpyridinium (18 µg/mL) (# p<0.01 vs CM).

To evaluate the implication of RAGE in calcification, we first tested the ability of AGE-BSA, a well characterized AGE, to induce vascular calcification in a model of elastocalcinosis in tissue culture that we previously developed [32]. However, this RAGE agonist was unable to induce calcification in this model. As it was previously demonstrated that the diffusion of a molecule in an intact tissue is closely associated with the molecular size [33], we postulated that the size of BSA probably limited its penetration.Thus, we used a smaller molecule, N-methylpyridinium, as a RAGE ligand. Since only one study demonstrated that RAGE was a receptor for N-methylpyridinium [31], we first sought to confirm that N-methylpyridinium can activate vascular RAGE signaling in our model. Knowing that RAGE signaling pathway includes activation of ERK, we measured ERK phosphorylation in VSMC incubated with N-methylpyridinium. A significant increase of p-ERK/ERK ratio was observed after cells were exposed to N-methylpyridinium (**Figure 3A**) and the use of an anti-RAGE antibody with N-methylpyridinium completely inhibited ERK phosphorylation (**Figure 3B**), suggesting that N-methylpyridinium indeed induces RAGE signaling pathways. No effect was found in the presence of a non-specific IgG (data not shown). Finally, primary VSMCs were exposed to N-methylpyridinium in a calcifying medium. Results presented in **figure 3C** demonstrate that N-methylpyridinium increased by 50% the calcification induced by the calcifying medium (**Figure 3C**). Overall, these results suggest that activation of RAGE signaling by N-methylpyridinium induced *in vitro* cell calcification.

**Figure 4:**
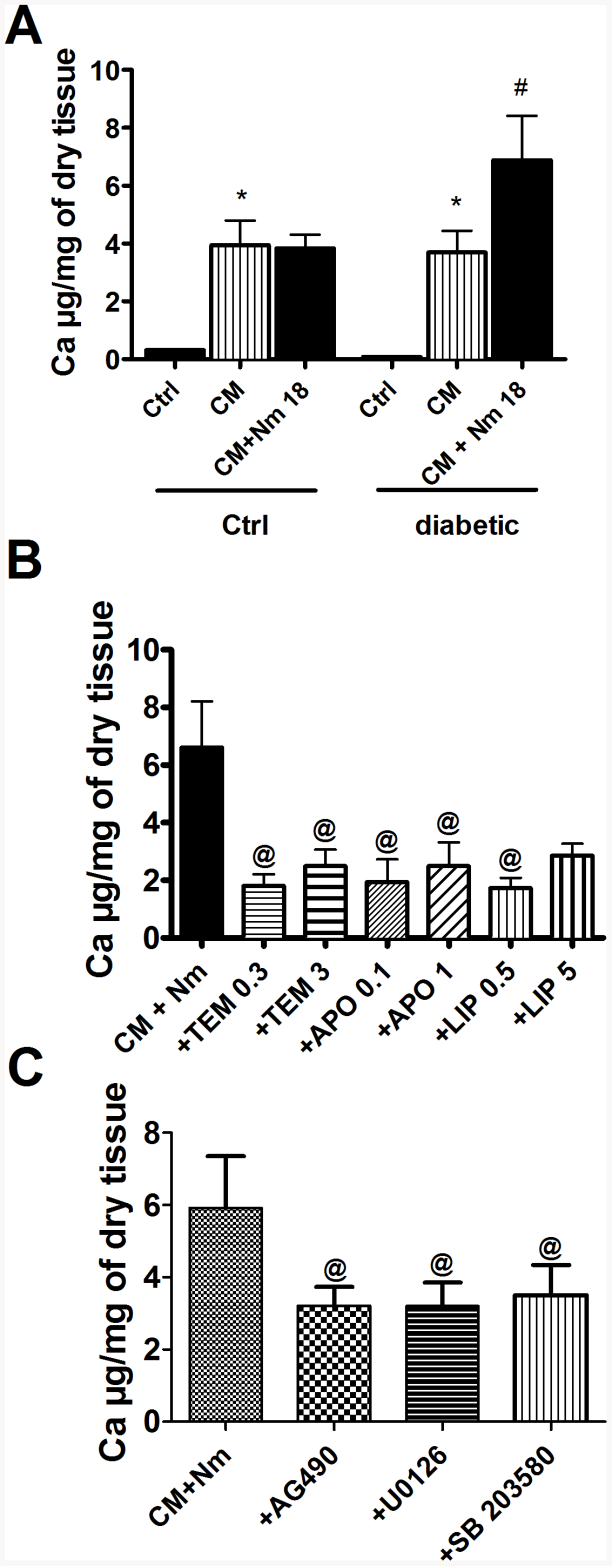
Implication of RAGE signaling pathways in femoral calcification induces by N-methylpyridinium. (A) Ex vivo calcification of femoral arteries harvested from diabetic and control rats and influence of AGEs receptor stimulation with N-methylpyridinium. CM: calcification medium; N-m 18: N-methylpyridinium 18 µg/mL. (* p<0.05 vs Ctrl; # p<0.05 vs CM). (B) Effect of different antioxidants on calcification stimulated with N-methylpyridinium, in femoral arteries of diabetic rats studied ex vivo. TEM: 4 hydroxy-tempol (0.3 and 3 mmol/L), APO: apocynin (0.1 and 1 mmol/L), LIP: alpha-lipoïc acid (0.5 and 5 mmol/L). (C) Effect of different pathway inhibitors used at 10 µM on calcification stimulated with N-methylpyridinium in femoral arteries of diabetic rats. AG490: JAK2 inhibitor, U0126: ERK1/2 inhibitor, SB 203580: p38 MAPK inhibitor. @ p<0.05 vs CM+Nm.

Next, in order to directly evaluate the impact of RAGE signaling induced by N-methylpyridinium on vascular calcification, we used our *ex vivo* model. We worked with streptozotocin-induced diabetic rats to have vascular tissue with molecular changes associated with diabetic conditions. Rats treated with streptozotocin presented significant hyperglycemia when compared to untreated rats used in the *ex vivo* experiments (D: 19.03±2.69 mmol/L vs Ctrl: 5.24±0.17 mmol/L, P<0.05). When femoral arteries were cultured in the calcification medium, there was no difference in calcification between untreated and diabetic rats. However, N-methylpyridinium enhanced calcium deposition in the femoral arteries of diabetic rats significantly but not in the femoral arteries of control rats (**Figure 4A**). Because the RAGE pathway is clearly associated with oxidative stress, we tested different anti-oxidants in the same *ex vivo* model. We observed that all three anti-oxidants used: 4-hydroxy-tempol, alpha-lipoïc acid and apocynin produced a significant reduction of calcification induced by N-methylpyridinium (**Figure 4B**). To better characterize the pathways implicated in the induction of calcification by N-methylpyridinium, we added different inhibitors of kinases potentially involved in the RAGE signaling pathway. The increase of calcification induced by N-methylpyridinium was significantly prevented by the use of AG 490, an inhibitor of JAK2, and of MAPK inhibitors U0126 and SB 203580 (**Figure 4C**).

**Figure 5:**
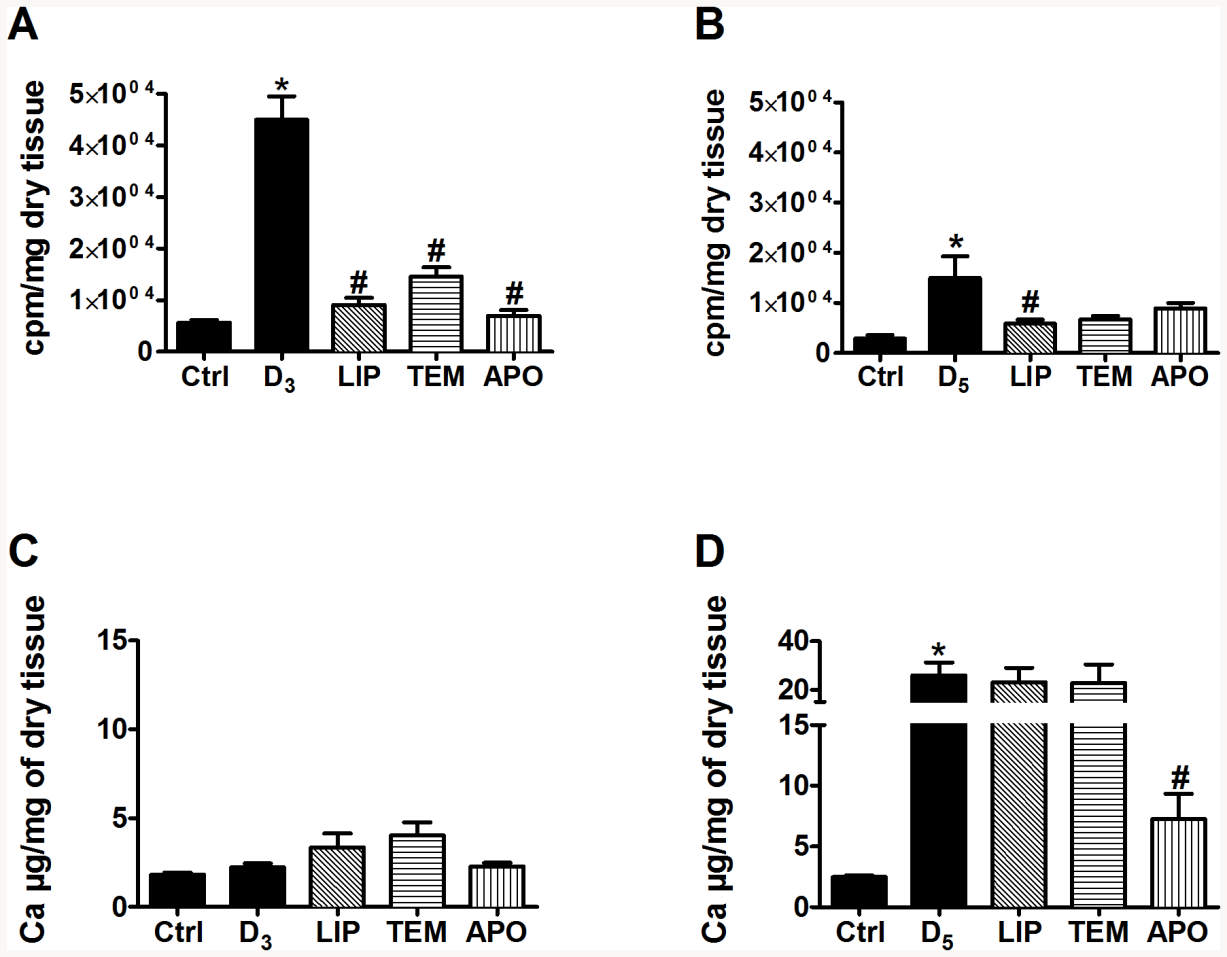
Impact of anti-oxidants treatment on femoral arteries. Lucigenin chemiluminescence after a 3-week treatment (A) and 5-week treatment (B). Calcium content after a 3-week treatment (C) and 5-week treatment (D). LIP: alpha-lipoïc acid (100 mg. kg-1. day-1); TEM: 4 hydroxy-tempol (20 mg. kg-1. day-1); APO: apocynin (2,5 mg. kg-1. day-1). (* p<0.05 vs Ctrl) (# p<0.05 vs D).

Next, the implication of ROS generation in calcification under diabetic conditions was studied by using anti-oxidants in our rat model of diabetes-accelerated medial calcification [25]. All groups of rats receiving a high fat diet and a low dose of streptozotocin demonstrated a significant hyperglycemia, confirming the induction of diabetes (data not shown). These rats were treated 3 to 5 weeks with 4-hydroxy-tempol, alpha-lipoïc acid or apocynin. Diabetes and WVK treatment were associated with increased superoxide anion production in femoral vascular wall (**Figures 5A**). This enhanced generation was more important in 3-week WVK treatment than in 5-week treatment (**Figures 5B**). We also observed an important enhancement of calcification in femoral arteries after 5-week WVK treatment (**Figure 5D**). Although all anti-oxidants reduced ROS production, the only anti-oxidant able to prevent significantly the acceleration of femoral calcification was apocynin (**Figure 5D**).

## Discussion

This study provides the first direct evidence of a decisive role of AGEs in the *in vivo* development of vascular calcification in experimental diabetes. Moreover, our results suggest the involvement of RAGE activation in the mechanisms leading to diabetes-induced vascular medial calcification. These results are particularly important in the context that diabetes is now frequently associated with aging. Thus, as noted in an editorial concerning our model [34], the use of an animal model of diabetes-accelerated medial calcification is an important tool to evaluate the impact of putative drugs on calcification induced in complex contexts.

In our diabetes model, we have previously demonstrated that calcification was related to the duration of diabetes [25]. In the present study, we measured the accumulation of AGEs and found that AGEs and calcium content followed a similar pattern and regional variations, both being more abundant in femoral arteries than in the aorta. Although previous studies in patients or on human tissues reported an association between AGEs and cardiovascular calcification [16], [35]-[37], an *in vivo* causal link remained to be determined. Our results with pyridoxamine and alagebrium are the first to establish the association between AGEs accumulation and medial calcification development. Indeed, we demonstrated that reduction of AGEs deposition induced by pyridoxamine and alagebrium is associated with a profound effect on vascular calcification. Like aminoguanidine, pyridoxamine inhibits AGE formation from Amadori products. However, pyridoxamine, in contrast to aminoguanidine, does not interact directly with Amadori intermediates, the reactive dicarbonyl intermediates [38], but interferes with post-Amadori oxidative reactions by binding catalytic redox metal ions [39]. Moreover, pyridoxamine is not an inhibitor of nitric oxide synthase, in contrast to aminoguanidine [40], and represents a better pharmacological tool. Alagebrium appears to break existing AGE-derived cross-links *in vitro* and *in vivo* in STZ rats [41]. Thus, pryridoxamine avoids AGE accumulation whereas alagebrium disrupts AGEs already accumulated. Accordingly, in our study, pyridoxamine prevented AGE accumulation, whereas alagebrium induced a regression of AGE cross-links. Therefore, we used the compounds with different protocols and the effect on calcium deposition was slightly different: pyridoxamine prevented calcium accumulation, while alagebrium blunted the progression of calcification. It did not produce a reversal of existing calcium, if we use the DWVK_3_ levels as an index of calcification before starting alagebrium. This suggests that AGE-derived cross-links are important in vascular calcification development, but not once calcification is set. Alternatively, other forms of AGEs that alagebrium cannot disrupt may also contribute to maintain the calcification response.

Although these results suggest that AGE inhibitors act by reducing AGE levels, it remains possible that the beneficial effects on calcification afforded by these inhibitors could involve a nonspecific antioxidant effect. Indeed, the ability of alagebrium to reduce protein kinase C expression and activity [42] raises that possibility given the well known implication of PKC in the generation of ROS [43]. Similarly, pyridoxamine also has pleiotropic effects such as scavenging carbonyl products and removing reactive oxygen species [44].

Calcification is one element contributing to arterial stiffness. It is expected that an improvement of medial calcification should reduce global stiffness. However, in our study, we did not observe changes in arterial stiffness with the anti-AGE treatments probably because no significant changes occurred in aortae prior to the beginning of the treatments. Indeed, PWV evaluates aortic stiffness, while the accumulation of calcium occurred mainly in the femoral arteries. Moreover, our study was designed to evaluate early events of arterial stiffness that are amplified by diabetes. At this stage, stiffness was not enhanced and we cannot conclude on the effect of the treatments. However, Wolffenbuttel *et al*. demonstrated a restoration of arterial compliance with alagebrium in STZ-induced diabetes [45]. Furthermore, the same therapeutic agent improved arterial compliance in aged patients with isolated systolic hypertension [19]. This could be explained by a reduction of cross-links, an improvement of endothelial function and, to a lesser extent, a reduction of fibrosis [46].

Medial calcification has been shown to increase in association with the accumulation of AGEs on elastin and collagen in the aorta of patients [36], [37] and in femoral arteries in our animal model. One of our aims was to elucidate the mechanism responsible for that association. *In vitro*, glycation of elastin enhances its binding to metal ions such as copper, iron [47] and calcium [17]. The calcium-binding activity was also increased on collagen exposed to glucose [37]. This could explain the correlation between calcification and AGEs. However, in our *ex vivo* model of medial calcification, we did not observe different levels of calcium accumulation in femoral arteries harvested from control and diabetic rats, suggesting that physical interaction between glycated proteins and calcium ions cannot be the only reason explaining the accelerated calcification in diabetes. Recently, some *in vitro* studies have suggested that AGEs could induce an osteogenic differentiation of smooth muscle cells followed by an active calcification process requiring the activation of RAGE signaling pathways [20]–[24]. It was also demonstrated that administration of AGEs increases vascular calcification [48]. Thus, AGEs not only enhance affinity of extracellular matrix components for calcium but also induce signaling pathways, resulting in phenotypic cell changes. In agreement, we demonstrated, in an *ex vivo* model of medial calcification, that stimulation of RAGE with N-methylpyridinium enhanced calcification in femoral arteries of diabetic rats but not in arteries of control rats. This difference could be explained by an increased expression of RAGE in diabetic rats, as observed in aortae of animals that received a STZ injection [48], [49]. Moreover, these results highlight the possible importance for diabetic patients to limit the consumption of RAGE ligands, as N-methylpyridinium that can be found in high amount in heated food (bread crust, coffee extract) [31], [50].

It is well known that stimulation of RAGE will activate many different pathways such as JAK, small GTPases (p21^ras^ and Cdc42) and mitogen-activated protein (MAP) kinases (activated by p21^ras^; p38 and ERK1/2) [51]–[54]. Our results demonstrated that inhibition of p38 MAPK significantly reduced calcification induced by N-methylpyridinium. These *ex vivo* results support the recent *in vitro* demonstration of the implication of p38 MAPK in calcification induced by AGE-RAGE interaction [21]. Our results also suggest a role for the ERK1/2 pathway in the calcification induced by the activation of RAGE, since the inhibition of ERK phosphorylation prevents the calcification induced by N-methylpyridinium. Accordingly, we recently demonstrated that ERK activation is implicated in medial vascular calcification [55]. We also demonstrated for the first time the implication of JAK2 in vascular calcification. Considering that RAGE is a member of the immunoglobulin family and the knowledge around the signaling events with these receptors, we can postulate that AGE-RAGE interaction causes the phosphorylation of JAK2 associated protein that subsequently activates the Ras/ERK1/2 pathway, with the probable implication of intermediate effectors, such as Raf and MEK. However, these results will require further testing to characterize more convincingly the link between the AGE-RAGE interaction and these signaling pathways.

It is well known that AGE-RAGE interactions enhance intracellular oxidative stress. The ROS produced by this interaction could have two origins: the NADPH-oxidase system [56] and/or the mitochondria [57]. Moreover, there is evidence showing that oxidative stress induces osteogenic gene expression leading to calcification of VSMC [58], [59]. Thus, we evaluated the implication of ROS production in the calcification induced by N-methylpyridinium. We demonstrated in *ex vivo* experiments that three different antioxidants have prevented mineralization induced by our RAGE agonist. In accordance, we showed that *in vivo* superoxide production preceded femoral calcification of diabetic animals, since ROS production was increased after 3 weeks while calcification became apparent only at 5 week. However, for an unknown reason, ROS levels decreased with the duration of treatment, maybe suggesting that it is a transient response. Another possibility could be that expression of endogenous antioxidant systems (SOD, catalase and glutathione reductase) could be enhanced between week 3 and 5, in response to the added stress. Although all antioxidants have reduced the level of superoxide found in femoral arteries, only apocynin significantly inhibited the *in vivo* calcification in those arteries, suggesting that ROS from NADPH oxidase activity is implicated in diabetic vascular calcification. It must be cautioned, however, that in a recent study, Heumuller *et al*. [60] showed that apocynin may not be an inhibitor of vascular NADPH oxidase but an important antioxidant. Although we cannot reject the possibility of a more general effect of apocynin, some studies have shown that the activity of NADPH oxidase is implicated in the expression of osteoblastic gene markers [58], [61]. More recently, two studies demonstrated that inhibition of NADPH activity prevent the calcification of VSMC expressing S100A12, a RAGE ligand [23], [24]. Importantly, we also recently demonstrated, with the use of siRNA against p22phox, that NADPH oxidase is involved in vascular calcification [55]. Since ERK1/2, p38 and JAK2 are ROS targets and have been previously associated with osteogenic differentiation of VSMC [9], [21], [58], the production of ROS induced by activation of NADPH oxidase could precede their activation and subsequently lead to vascular calcification. It is also possible that RAGE directly activates these pathways. Thus, more studies are mandatory to clearly determine the mechanism by which RAGE agonist-induced ROS production amplifies calcification.

The implication of ROS in vascular calcification was demonstrated by many studies. However, the use of antioxidant does not necessarily guarantee a protection against vascular calcification. This was demonstrated by the inefficiency of tempol to prevent the calcification of aortic valves [62]. Moreover, our results also showed that tempol and LIP had no impact on calcification despite the reduction of the superoxide levels. This may suggest that scavenging free radicals is not sufficient in vivo, and could require inhibition of production at its source, or different antioxidants could have undesired effects cancelling the direct impact on calcification. For example, LIP can induce p38 MAPK [63], [64] and ERK1/2 [65], two signaling pathways associated with calcification. Nevertheless, more studies are needed to clarify the mode of action of the various antioxidants and to determine the ideal target to reduce oxidative stress and prevent vascular calcification.

In conclusion, we demonstrated a crucial role of AGEs in medial calcification in the context of diabetes. This could represent a mechanism by which diabetes accelerates calcification and vascular stiffness. Thus, drugs that can influence AGEs, such as alagebrium, could be of therapeutic benefit, especially in the early phase of the disease process, when calcification seems to occur. Our results also suggest that part of the AGE-induced calcification could represent a response to AGE-RAGE interactions. Drugs affecting the binding of AGEs or the signaling elements leading to the final response, such as NADPH inhibition, could also be of interest to limit the vascular complications of diabetes.
